# Hypoxia Signaling in Cancer: From Basics to Clinical Practice

**DOI:** 10.3389/pore.2021.1609802

**Published:** 2021-05-03

**Authors:** Anna Sebestyén, László Kopper, Titanilla Dankó, József Tímár

**Affiliations:** ^1^ 1st Department of Pathology and Experimental Cancer Research, Semmelweis University, Budapest, Hungary; ^2^ 2nd Department of Pathology, Semmelweis University, Budapest, Hungary

**Keywords:** cancer, hypoxia, angiogenesis, metabolism, therapy

## Abstract

Cancer hypoxia, recognized as one of the most important hallmarks of cancer, affects gene expression, metabolism and ultimately tumor biology-related processes. Major causes of cancer hypoxia are deficient or inappropriate vascularization and systemic hypoxia of the patient (frequently induced by anemia), leading to a unique form of genetic reprogramming by hypoxia induced transcription factors (HIF). However, constitutive activation of oncogene-driven signaling pathways may also activate hypoxia signaling independently of oxygen supply. The consequences of HIF activation in tumors are the angiogenic phenotype, a novel metabolic profile and the immunosuppressive microenvironment. Cancer hypoxia and the induced adaptation mechanisms are two of the major causes of therapy resistance. Accordingly, it seems inevitable to combine various therapeutic modalities of cancer patients by existing anti-hypoxic agents such as anti-angiogenics, anti-anemia therapies or specific signaling pathway inhibitors. It is evident that there is an unmet need in cancer patients to develop targeted therapies of hypoxia to improve efficacies of various anti-cancer therapeutic modalities. The case has been opened recently due to the approval of the first-in-class HIF2α inhibitor.

## Introduction

**FIGURE 1:**
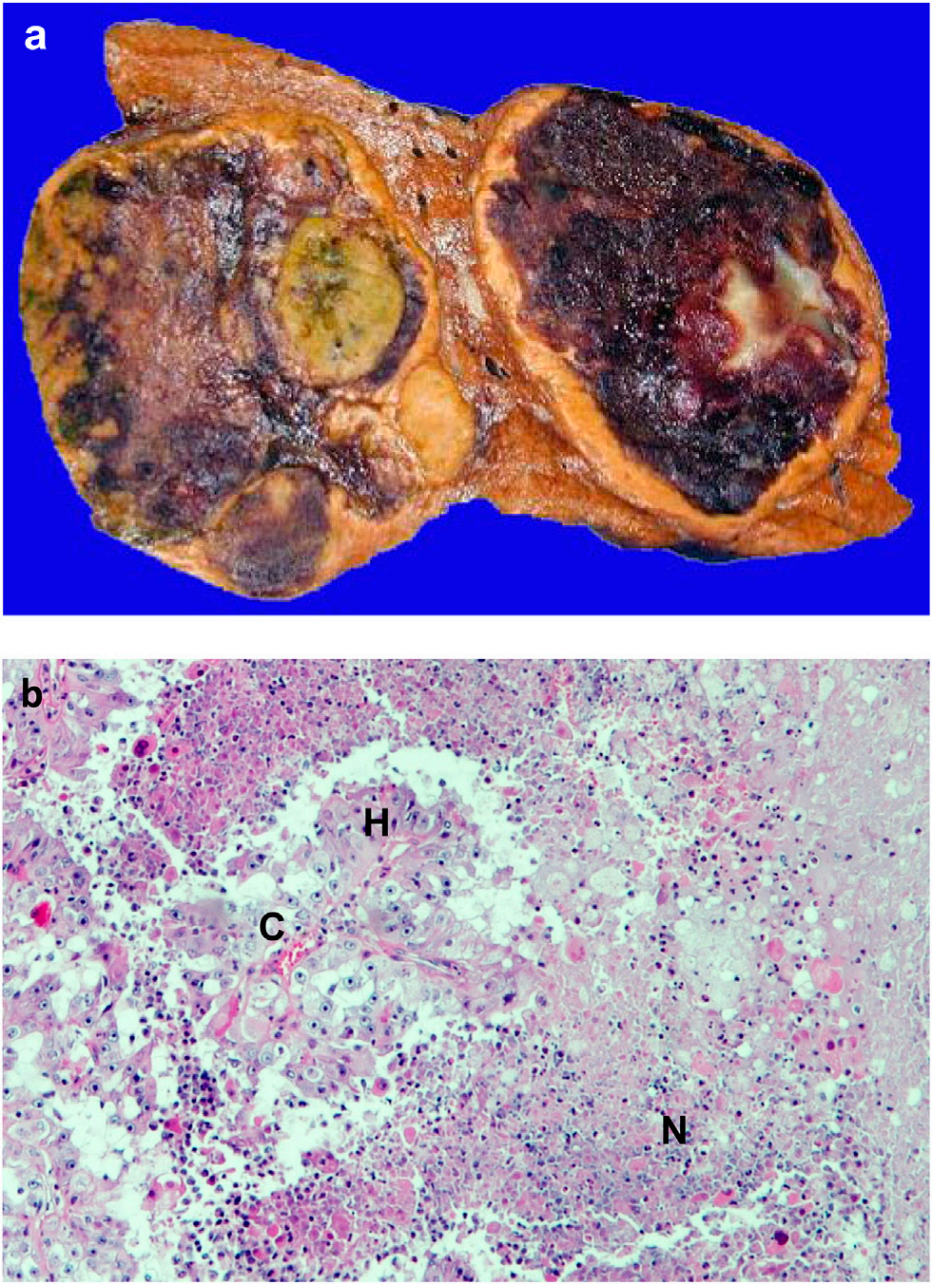
Necrosis in cancer. **(A)**. Macroscopic picture of hemorrhagic necrosis in liver cancer; **(B)**. Microscopic picture of necrosis in renal cell cancer (HE staining). C = capillary, H = hypoxic area, N = necrotic area.

One of the most typical macroscopic pathologic characteristics of malignant tumors is the presence of bleeding and necrosis (Figure 1). This is due to the fact that growth of normal tissues manifests in harmony with vascularization while in case of cancer, tumor growth is driven by activated oncogenes irrespective of permissive vascular supply. Necrotic tumor tissue does not present much harm to the host organism, but the hypoxic part of the tumor tissue and the hypoxic tumor cells are usually the major drivers of tumor progression [1–3].

There is a difference in the oxygenation/physoxia of various normal tissues (4–10% O_2_) due to the differential blood supply and tolerance to hypoxia. The most oxygenized tissues are renal cortex, liver, breast and pancreatic tissues, while the least oxygenized ones are brain, lung and intestinal mucosa (Table 1). As compared to normal tissues, oxygenation levels are much lower in cancers, even in the most vascularized tumors the O_2_ rate is only 2% (lung cancer), but in most cases it is much lower, especially, for pancreatic cancers, where this rate is the lowest (0.3%) [1].

**TABLE 1:** Comparison of oxygenation levels in cancer and host tissues. [1].

Cancer	% O_2_	Host tissue	% O_2_
Lung cancer	2.2	Renal cortex	9.5
Rectal adenocarcinoma	1.8	Breast tissue	8.5
Glial tumors	1.7	Pancreatic tissue	7.5
Breast adenocarcinoma	1.5	Liver	7.3
Renal cell cancer	1.3	Lung	5.6
Cervical squamous cell cancer	1.2	Uterine cervix	5.5
Hepatocellular cancer	0.8	Brain	4.6
Pancreatic adenocarcinoma	0.3	Rectal tissue	3.9

For individual references see [1].

Hypoxia has various forms: acute, chronic, toxic and systemic ones. In cancers, toxic form of hypoxia is not significant. Acute hypoxia is perfusion hypoxia while chronic hypoxia is characterized as diffusion hypoxia, indicating various pathomechanisms behind. Systemic hypoxia in cancer patients is also a frequent event. Collectively, cancer hypoxia usually is a combination of acute, chronic and systemic forms of hypoxia, which not only drives tumor progression, but also a leading cause of resistance to various therapeutic modalities [3–6].

Below we will provide an overview about the causes of cancer hypoxia and the induced cellular responses; and additionally, we will also summarize the metabolic and immunological consequences. At the end, current therapeutic approaches to overcome cancer hypoxia will be summarized.

## Pathomechanism of Cancer Hypoxia

**FIGURE 2:**
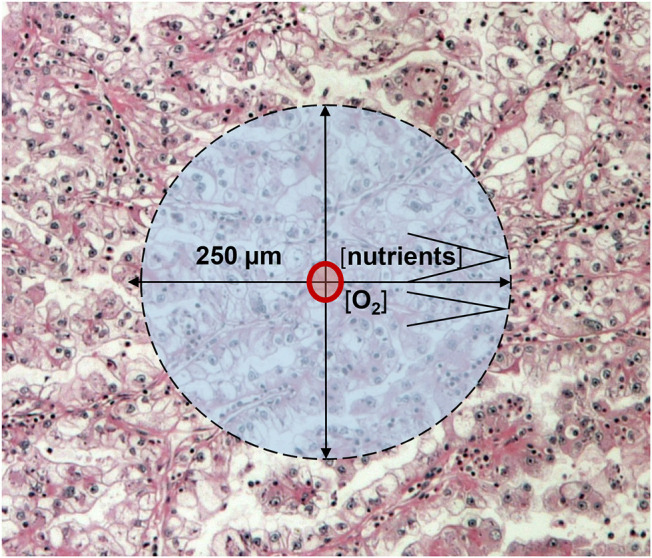
Schematic presentation of cancer growth beyond 1 mm^3^: oxygen and nutrient diffusion distances.

According to Folkman’s theory, a tissue (including cancer) which growth beyond 2–3 mm^3^ requires new blood vessels [2]. We now know that oxygen and nutrient supplies are considered to be optimal in a 250-μm radius of capillaries in various tissues, accordingly a >1 mm^3^ tumor tissue can survive without new vessels (Figure 2). Since cancer growth exceeds that size, cancer progression/development is driven not by the presence of blood vessels but the immanent oncogenic mechanisms. In cancers, it is almost inevitable that hypoxia would develop which can be due to: 1) compressed intratumoral vessels [3], 2) abnormal newly developed intratumoral capillaries [4], or 3) the systemic hypoxia in the host. Acute hypoxia without resolution leads to the development of necrosis in case of extremely low O_2_ levels which is not normalized rapidly at that area. However, chronic hypoxia is the most typical form of hypoxia in tumor tissues [5]. Meanwhile, acute and chronic hypoxia are combined in frequently in cancer tissues leading to central tumor necrosis and surrounded by hypoxic areas (Figure 1B). Chronic hypoxia may induce physiological responses in tumor tissue, but in case of genetic changes of the signaling pathway components, this response could be profoundly different [6].

## Oxygen-Dependent HIF Activation

**FIGURE 3:**
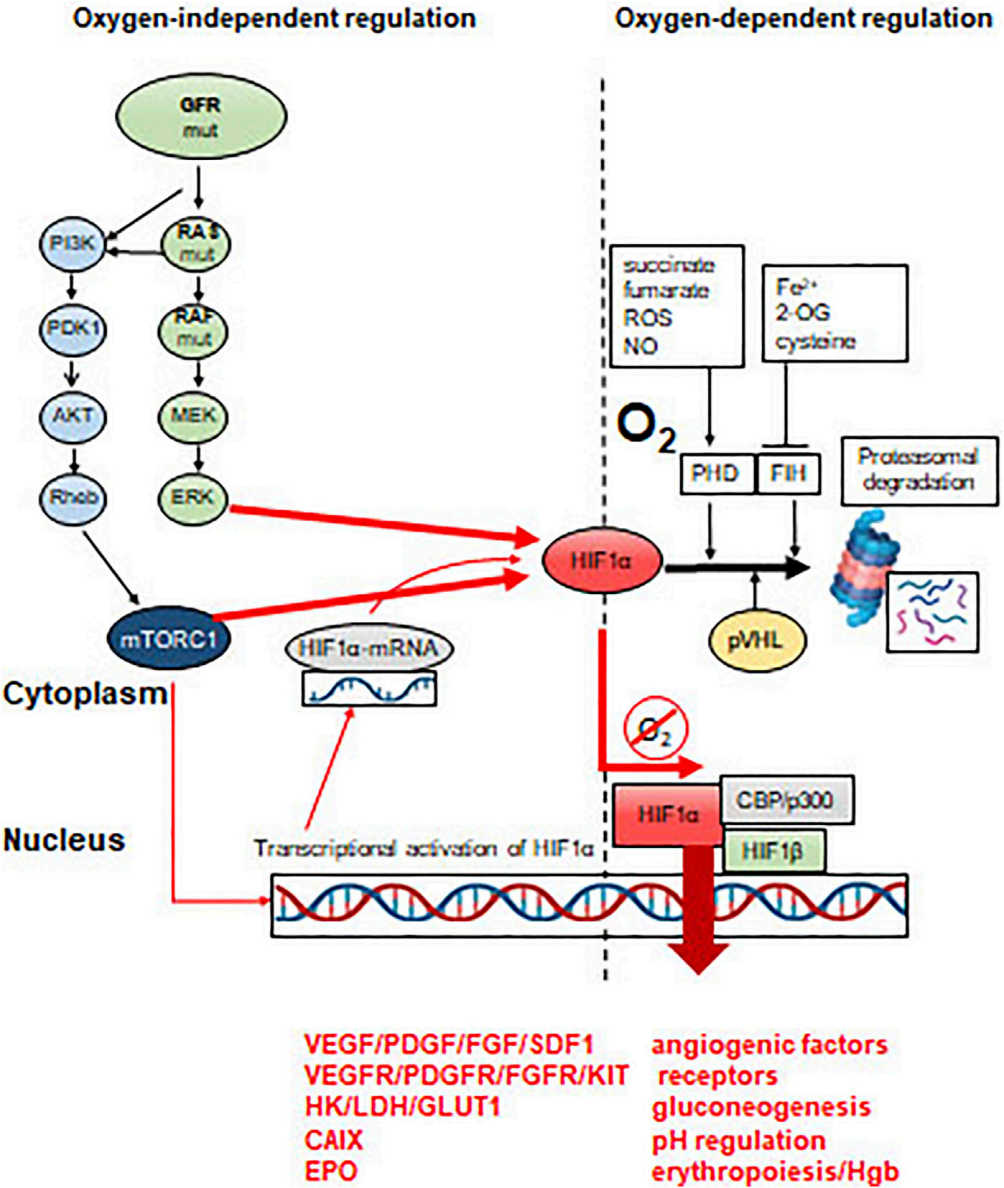
Molecular mechanisms of activation of HIFα transcription factors. HRE = hypoxia-responsive element in the promoter region of specific genes. Effect of constitutive oncogenic activation on HIFα. Proteasomal degradation is inhibited by mTOR or ERK activity, even in the presence of sufficient oxygen levels.

In (cancer) cells there are two O_2_ sensors: the prolyl-hydroxylases (PHD1-3) and the asparaginyl-hydroxylase (FIH), characterized by different O_2_ affinities (low: PHD, high: FIH). Accordingly, PHD activity is decreasing linearly with lowered O_2_ levels, while FIH activity would decrease only at very low O_2_ levels. A unique role of the oxygen sensors is to hydroxylate HIFα transcription factors. At high O_2_ levels, hydroxylases label HIFα proteins for VHL, which recognizes these forms and send them for proteasomal degradation by recruiting ubiquitin ligases (Figure 3). In this way HIFα proteins are characterized by the shortest half-life among cellular proteins. HIF proteins are α/β heterodimer transcription factors where the expression of the HIFβ partner is constitutional, but it is inactive as a monomer. This powerful transcription factor system is under strict regulatory control: at normoxia, prolyl-hydroxylation ensures protein degradation, while aryl-hydroxylation results in functional inactivation due to the inhibition of coactivator bindings (p300/CBP). Low O_2_ levels stabilize HIFα proteins which accumulate and translocate to the nucleus. In parallel, it also activates the expression of certain genes which are involved in adaptation to hypoxic conditions [5, 7] (Figure 3).

There are 216 genes in the human genome which contain HIF-responsive elements (HRE) in their promoters [6, 8, 9] However, the list of classical HIF regulated genes is much shorter (∼40) [9]. (Table 2). These target genes are responsible for the cellular responses to hypoxia or the accommodation to chronic hypoxia. Meanwhile, the best-known HIF-target genes are angiogenic factors (e.g. VEGF, FGF, PDGF, ANGP1/2 and SDF1), angiogenic factor receptors (VEGFR2/KDR, VEGFR1, KIT) or the O_2_ transport capacity regulator, EPO [6]. Those genes which are involved in hypoxia-induced metabolic adaptation are equally important (see later). HIF1α and HIF2α have different target gene profiles, but their regulation and accordingly their roles can be different in cancer progression.

**TABLE 2:** Classical HIF1A regulated genes based on key publications [6, 9].

ADM	CDKN1A	FLT1	LDHA	PKM	TPI1
AK3	CITED2	GAPDH	MDR1/ABCB1	SERPINE1	VEGFA
ALDOA	CP	HK1/2	NOS2	SLCA1/3	
ALDOC	EDN1	HMOX1	P4HA2	TF	
BNIP3	ENO1	IGF2	PFKL	TFRC	
CAIX	EPO	IGFBP1/2/3	PGK1	TGFB3	

It is a unique consequence of cellular hypoxia that the induced metabolic changes result in production of reactive oxygen species (ROS) which can induce DNA damages, similar to other mutagens. Since under hypoxia the function of DNA repair enzymes can be downregulated, there is a risk of further accumulation of DNA mutations in oxygen-deprived conditions [10].

The vital role of HIF transcription factors is reflected by the fact that their mutations are very rare in cancers: at low frequency, HIF1α mutation can be detected in renal cell cancer [11], while HIF2α mutation results in the development of a rare tumor, paraganglioma [12]. Mutations of HIF regulators–such as VHL, even in the form of germline ones (von-Hippel Lindau syndrome)–are much more frequent in cancers [13]. The consequences of the constitutive HIF activation during development can be observed in VHL syndrome where hemangioblastomas, neuroendocrine tumors of the pancreas and adrenal gland may develop beside the characteristic renal cell cancer. Accordingly, the genetic prototype of HIF-deregulated cancer is the sporadic renal cell carcinoma, where the incidence of the loss of function mutation of VHL is 50%, resulting in an angiogenesis-dependent tumor [14].

Detection of hypoxia in human tumors is a challenge due to the fact that tissue fixation can alter HIF- and hypoxic target protein detections, moreover, native specimens with preserved O_2_ supply are not readily available. Meanwhile HIFα detection in combination with CAIX, GLUT1 or VEGF can help to overcome this problem [15]. Especially, the combination of mRNA and protein detection of HIF1/2α and their targets could be useful. Using such a combined approach in metastatic renal cell cancer, it was possible to demonstrate that high HIF1α and low HIF2α expressions or a “HIF-index” are poor prognostic factors, when CAIX, GLUT1 and GAPDH overexpressions follow this prognostic trend [16].

## Oxygen-Independent HIF Activation: “Pseudohypoxia”

Mutations of growth factor receptors or members of their respective signaling pathways are characteristics for many different cancer types: EGFR mutations in lung adenocarcinoma, HER2 amplifications in breast and gastric cancers, RAS mutations in lung and colorectal cancers, BRAF mutations in melanoma, thyroid or GI-tract cancers or mutations of the lipid kinase signaling pathway members (PI3KCA, AKT) in various cancer types [17]. One of the common functional consequence of these activating mutations is the extreme activity of mTORC1, resulting in constitutive protein synthesis and/or stabilization/functional activation of HIFα. On the other hand, HIFα stabilization can also be the consequence of the increased activity of the RAS-MEK-ERK signaling pathway [18]. Connection among tumor hypoxia, increased HIFα activity and tumor progression is a dogma today [6]. In hypoxic tumor cells, HIF activates several HRE genes that are essential for migration, invasion and metastasis: for example, autocrine motility factor and its receptor (AMF and AMFR); MET oncoprotein, receptor for scatter factor (paracrine regulators); CXCR4 chemokine receptor, matrix metalloproteinases such as MMP2/9 and collagen network remodeling lysil- and prolyl-hydroxylases (LOX and P4HA). The increased migratory activity of hypoxic tumor cells is also due to the activation of the RhoA/ROCK1 signaling pathway leading to cytoskeletal remodeling [6, 18, 19].

## Vascularization of Hypoxic Cancer Tissue

During chronic inflammation or tissue necrosis in the regenerating normal tissues, development of novel capillary network takes place in the form of neo-angiogenesis. This process is fundamentally different from the embryonic development of blood vessels, called vasculogenesis, referring to the fact that in the developing tumor tissues there was no vasculature previously. This later process is based on the mobilization of angiogenic precursors which migrate from bone marrow to developing tissues. In the new location, these cells differentiate into endothelial cells which than form new vasculature in cooperation with mesenchymal cells [20]. In cancer tissues, hypoxia or oncogenic activation of the HIF pathway induces the expression of genes involved in angiogenesis (VEGF, PDGF, SDF) to increase the blood supply and O_2_ level. The production of large amount of angiogenic factors and/or cytokines by tumor tissues can also support the migration of these precursors from the bone marrow into the tumor tissue. However, the contribution of vasculogenesis to the vascularization of tumor tissue is minimal in humans [21].

**FIGURE 4:**
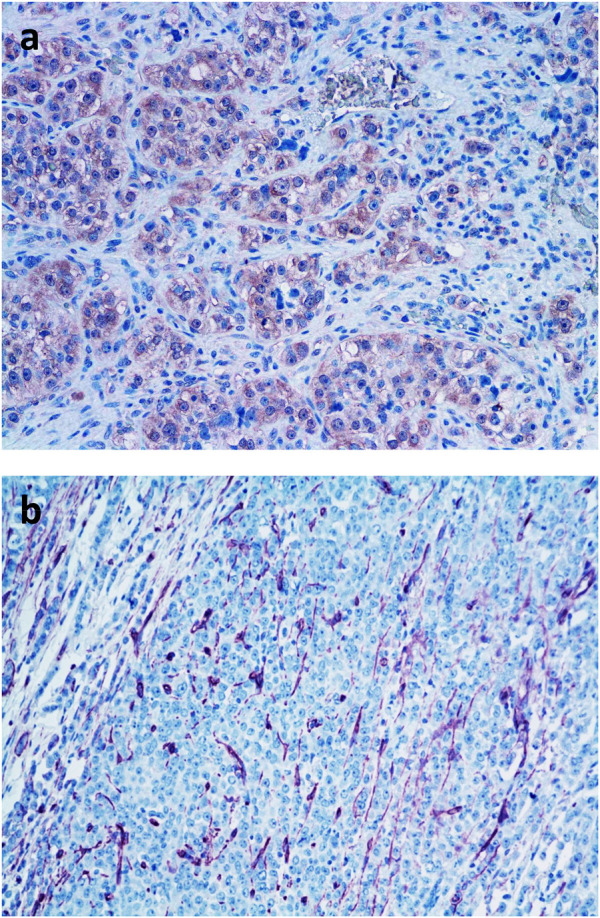
Demonstration of intratumoral microvasculature in breast cancer. **(A)**. Detection of VEGF in tumor cells by immunohistochemistry (pink color); **(B)**. Neo-angiogenesis in breast cancer tissue: demonstration of intratumoral blood vessels by immunohistochemical labeling of CD31 positive endothelial cells (pink color) BAR = 100 μm.

It is much more characteristic in tumor tissues that the production of angiogenic factors/cytokines induces “regeneratory”-type of neo-angiogenesis. In this case, the new capillaries are derived from pre-existing peritumoral capillary network in the form of sprouting (Figure 4). Tumor-induced neo-angiogenesis is initiated by local production of VEGF, PDGF, FGF, TGFβ, TNFα, and ΑΝG2. This type of neo-angiogenesis occurs at the venous site of the capillary network [20]. It is still widely accepted that these new capillaries are growing into the developing tumor tissue [2, 20]. However, it is much more common that the tumor-induced novel peritumoral capillary network is incorporated into the tumor tissue by vessel cooption [20, 22, 23]. Furthermore, this reparative neo-angiogenesis takes place in a host tissue specific manner in various tumors [24, 25].

It is more and more evident that reparative neo-angiogenesis is not the most common mechanism to provide blood supply for tumor tissues. Experimental and clinicopathological data demonstrated that the vessel-cooption (incorporation of preexisting vessels) is the most conventional form of vascularization of primary and metastatic tumor tissues. The capillary density of certain tissues (e.g. lung, liver) is sufficiently high, fulfilling the requirement of 1-mm^3^ size for tumor growth [2, 20, 23]. Drivers of this type of vascularization are tumor-derived cytokines involved in endothelial cell survival such as angiopoetins and VEGF [23–25]. There is another non-neoangiogenic form of blood supply of tumor tissues (especially in brain tumors or brain metastases), the glomeruloid vasculogenesis/angiogenesis [20, 26], where the preexisting capillaries are remodeled into novel chaotic, tortuous capillary loops. Major drivers of this remodeling are the extremely high local concentrations of VEGF, FGF and PDGF complemented by CSF1, SDF1 and SCF1.

There is a fundamental alteration of gene expression regulation in cancer cells which can provide stem cell properties. This aberrant regulation can result in the loss of linage-specific gene expressions and acquiring new ones. The best-known example of this alteration is the epithelial-mesenchymal transition (EMT) [27]. But there are other forms of such transitions (mimicries) such as 1) neurogenic mimicry, expression of neurogenic genes in non-neural cell types, mostly epithelial cells; 2) megakaryocytic mimicry, expression of megakaryocyte-specific genes in non-bone marrow cell types [28]; or 3) vasculogenic mimicry*,* [29–31] expression of endothelial genes in non-angioblastic cells, also driven by chronic hypoxia and/or constitutive HIFα expression. If tumor cells express endothelial genes, it can result in the development of novel phenotypic features, communication ability between endothelial cells of the preexisting capillaries and tumor cells which form capillary lumina connected to the blood capillaries [30, 31].

It is of note, that vascularization of tumor tissue is cancer-type specific and greatly depends on the host tissue. Accordingly, it can be different in metastases as compared to primary tumors [24, 25].

Microvessel density of tumor tissues are usually high and it is expected that the blood supply of tumors is also optimal. In contrast to this presumption, tumor tissue is hypoxic. One reason of the poor blood supply is that the interstitial pressure is increased in tumor tissues resulting in the collapse of tumoral capillaries [3]. Another feature of the tumoral blood vessels (newly developed or incorporated) is that their structure is abnormal, the endothelial lumen is leaky and/or the supportive pericytes are missing [4]. In this way, a vicious cycle develops: the hypoxic tumor becomes angiogenic and tries to develop or coopt more capillaries, but this do not lead to higher O_2_ levels; on the contrary, tumor tissue hypoxia is stabilized. According to Table 1, the best oxygenized tumor is lung cancer closely followed by breast and rectal cancers. However, kidney cancer is characterized by the highest microvessel density followed by lung or breast cancers [32], suggesting that there is no direct connection between vascularization and oxygenation of cancer tissues.

Even if tumor tissue would be optimally vascularized, systemic hypoxia could also lead to tumor tissue hypoxia in cancer patients. There are several causes for systemic hypoxia such as bleeding, hemolysis (hemolytic anemia), bone marrow infiltration by tumor cells, bone marrow depletion by chemo-or radiotherapy, nephrotoxicity of chemotherapy, chronic obstructive lung disease or cardiac failure. Accordingly, systemic causes of tumor tissue hypoxia are outstanding features of malignant tumors [33].

## Metabolic Consequences of Tumor Hypoxia

Biological oxidative processes (oxidative phosphorylation) can be termed as the bioenergetically optimal energy productions in cellular metabolism. In hypoxic or pseudo-hypoxic conditions, cancer cells with rapid proliferation capacity require high energy and nutrient supply, and the related bioenergetic pathways have to be re-wired [5]. In hypoxic microenvironment, HIF activation results in the accumulation of lactic acid (as a consequence of anaerobic glycolysis) which is a characteristic metabolic feature of the majority of tumors. Furthermore, in cancer cells independently of the oxygen concentration changes, the erobic glycolytic phenotype (Warburg effect–aerobic glycolysis) can also be found [34].

**FIGURE 5:**
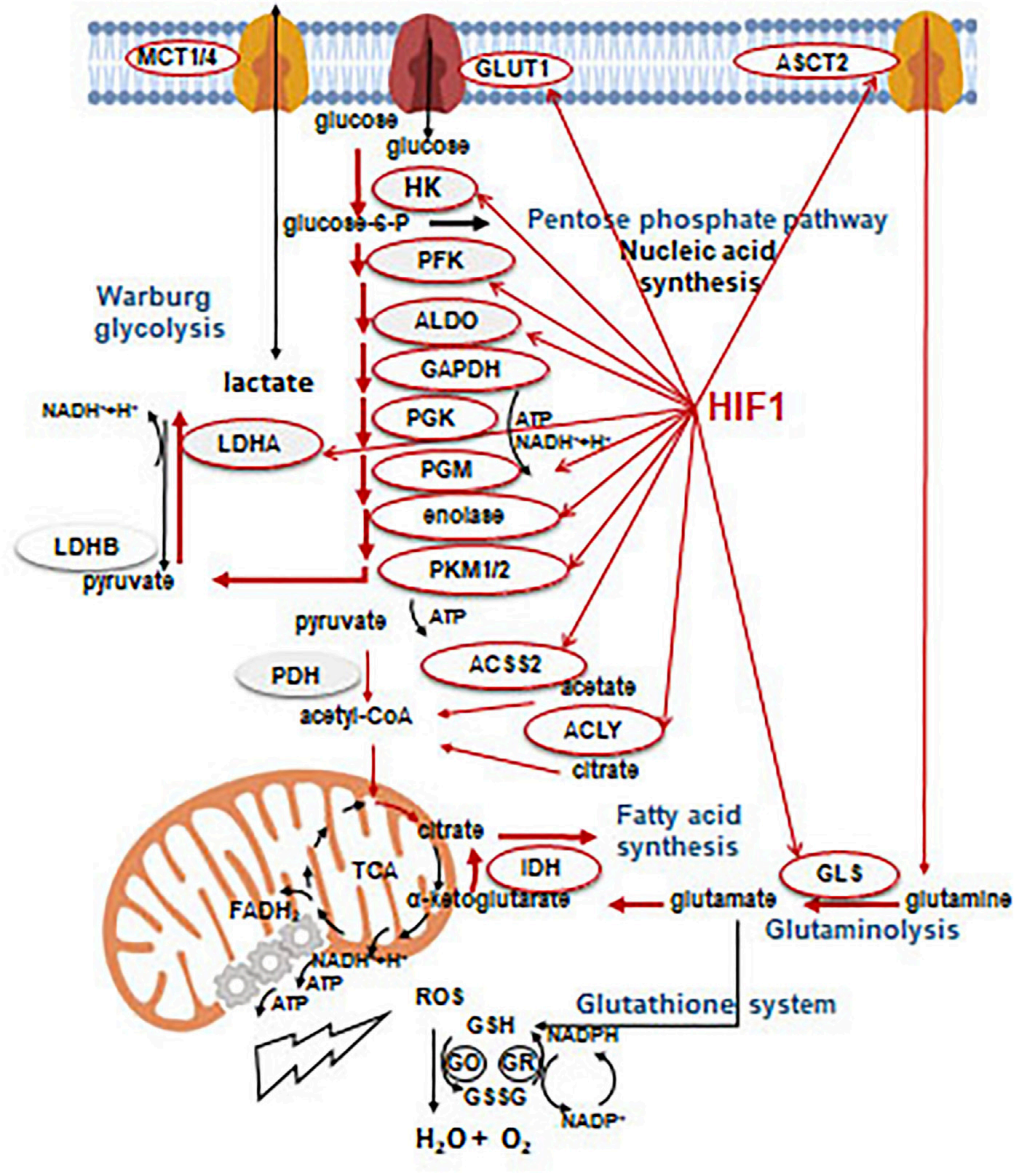
Effects of HIF1α on the metabolic rearrangement. Without going into details, enzymes and processes which can be controlled and/or associated with glycolytic phenotype during metabolic rearrangement by HIF1α (regulation). Beside the effects on HIF1α targets involved in metabolic, glycolytic rearrangement (narrow red arrow), the most frequent and significant metabolic shifts (thick red arrow) are also presented in the figure.

It has been well-known since the early 2000s, that the HIF1α stabilization and the elevated HIF1α protein levels are characteristic for ∼50% of tumor cells under normoxia [35]. As a consequence, the productions of several glycolytic enzymes or transporters increase in malignant cells, e.g. the gene expressions of glucose transporter (GLUT1-3) or pyruvate-dehydrogenase-kinase 1 (PDK1), pyruvate kinase isoform 2 (PKM2) are elevated. These contribute to the conversion of pyruvate to lactate. In addition, entering acetyl-CoA into the tricarboxylic acid cycle (TCA) can be inhibited, leading to decreased mitochondrial respiration and oxygen consumption. In parallel, glutamine or other intermediates of various metabolic processes can fuel TCA with anaplerosis [3], e.g. the increase in glutaminase expression, glutaminolysis or influencing protein and lipid metabolism (Figure 5).

HIF1α-mediated metabolic rearrangement can also contribute to other microenvironmental alterations since lactate production and acidification are important oncogenic features. Based on these, the most important oncogenic impact of HIF1 activation is necessarily the angiogenic effect. HIF1α has a comparable importance in maintaining the proliferation demands for rapid metabolic rearrangements [34]. In tumor microenvironment, the energy and nutrient demand of tumor cells can dominate but the tissue oxygenation could also affect the metabolism of stromal cells. In non-transformed cells of well-oxygenated tissues and even in tumor cells which are located near to blood vessels, an oxidative phenotype, the reverse utilization of lactic acid (reverse Warburg effect) can be observed [36]. The evolving metabolic symbiosis can guarantee the optimal utilization of energy resources at tissue level [37].

The most important differences in metabolic machinery of tumors and their normal equivalents are mutations of key genes, and the related signaling pathways which drive continuously high metabolic activity for proliferation. This can be emerged concomitantly with proper net lactic acid overproduction and metabolic flexibility [38, 39]. It is also known that certain tumors can have differences in basic metabolic processes e.g. alterations in anaplerotic mechanisms of TCA (e.g. lipid metabolic alterations or autophagy). Even certain oncogenic mutations result in oncometabolite production [40]. Tumor growth induces activation of several additional early or late stress responses i.e. elevation of ROS supporting hypoxia, extracellular acidification to maintain optimal energy level in nutrient or oxygen deprivation. Simultaneously, other metabolic adaptation mechanisms can induce the activation of antioxidant processes in cancer cells. Glutathione system is one of the important antioxidant cellular programs. Glutathione reductase neutralizes H_2_O_2_ with glutathione derived from cysteine, glutamate and glycine. In a further step, NADPH, as a cofactor of glutathione oxidase, converts glutathione. Thioredoxin system, as an additional alternative, can reduce the H_2_O_2_ level by the use of NADPH. In summary, NADPH has a remarkable role not only in biosynthetic processes but also in buffering ROS levels [41] (Figure 5). The balance of ROS-regulating capacities is also an important element of metabolic changes, which foster hypoxic processes. Recently, alterations in several tumor-specific factors have been characterized in the regulation of ROS generation such as NRF2 or SLC7A.

mTOR kinase is an important regulatory element of signaling network and metabolism has special and context-dependent role in hypoxia-related cellular events. The two different mTOR complexes have critical functions in cellular homeostasis by sensing and synthesizing intra- and extracellular conditions [42]. Moreover, mTORC1 influences the protein expression level of several onco-proteins such as HIF1α at post-transcriptional, translational level. However, the production of HIF1α requires mTORC1 activity, additionally, post-translational degradation of the protein regulated directly and quickly by O_2_ level [43]. Therefore, mTOR hyperactivity provides stabilization of HIF1α protein and other regulatory failures contributing to the HIF1α stabilization in pseudohypoxic tumor tissues [44]. Moreover, other cellular stresses (nutrient deprivation, DNA damage responses, low energy level and “real” cellular hypoxia) could reduce mTORC1 activity and slow down the tumor proliferation/growth. This situation rewires cellular metabolic processes e.g. reduces oxygen consumption and/or induces autophagy [45] leading slower metabolic activity and forces cellular survival mechanisms with balanced bioenergetics [46]. They could give an opportunity to restore cellular homeostasis and mTORC1 activity maintaining pseudohypoxia in tumor cells.

Considering the fundamental regulatory changes of certain tumors (mutations of oncogenes/tumor suppressors in signaling) and their metabolic consequences or the expected effects of currently available treatments, novel therapeutic options could be introduced to target and inhibit metabolic adaptation mechanisms. The importance of the latter is that hypoxia-induced metabolic processes (e.g. in case of anti-angiogenic treatments) have to be taken into account since using inhibitors of metabolic adaptation regulators (such as mTOR) or other enzymes, metabolic catastrophe, synthetic lethality could be induced in cancer cells [47, 48].

## Immunologic Consequences of Tumor Hypoxia

Antitumoral immune responses are affected by (tumor) tissue hypoxia [40]. Antitumoral innate immune responses are mediated by NK cells and macrophages. It is important that macrophages are sensitive to hypoxia and instead of the M1/antitumoral polarization in normoxia, under such circumstances they obtain M2/immunosuppressive phenotype [49]. Furthermore, HIF activation in hypoxia enhances immune suppressive effects of myeloid-derived suppressor cells [50]. On the other hand, NK cells remain active against tumor cells in hypoxia [51].

Hypoxia fundamentally affects the acquired immune responses to tumors as well. One of the main immunosuppressive cytokine in the hypoxic microenvironment is VEGF [52]. In hypoxia, VEGF promotes CD4^+^ T-cell differentiation into T-regulatory cells, suppressing the emerging immunoreactions. Hypoxia modulates immune checkpoint inhibitory molecules on infiltrating cells and tumor cells by inducing PDL1 expression. In CD8^+^ cytotoxic T-cells, hypoxia also induces CTLA-4 receptor, another checkpoint regulator [53]. In lung cancer, it was demonstrated that cancer cells overexpress PDL1 at the border of necrotic tumor while the infiltrating cells express PD1 and activate immunosuppressive mechanisms [54]. For an effective antitumoral response, the density of immune effector cells is an important parameter. It was shown in skin melanoma that increased tumoral vascular density is associated with increased macrophage and T-cell density [55].

## Clinical Imaging of Tumor Hypoxia

It is a longstanding goal in experimental cancer research and clinical oncology to develop reliable markers to measure pO_2_ levels in cancer tissues or to detect hypoxia. It is an invasive approach to use polarographic oxygen electrodes to measure pO_2_ levels in cancer tissues. On the other hand, there are immunohistochemical techniques to asses tissue hypoxia in biopsies. This is also an invasive technique since it requires to inject exogenous hypoxia marker into the tumor tissue before resection such as pimonidazol or a derivative, EF5. It is a less reliable approach to use endogenous hypoxia markers such as HIF1α or GLUT1 immunohistochemistry. The problem here is that not only hypoxia can induce the expression of those marker genes but also various genetic changes of the tumor which cause overexpression of the markers independent of the hypoxia.

Accordingly, non-invasive techniques have been developed and tested clinically. One approach is to use magnetic resonance imaging such as blood-oxygen level dependent imaging (BOLD) able to monitor tissue perfusion. On the other hand, nuclear magnetic resonance spectroscopy can be used to measure increased lactate or decreased ATP levels in cancer tissues: unfortunately, the sensitivity and resolution of these techniques are very low. The gold standard technology to measure tumor tissue pO_2_ levels is PET using ^15^O_2_. However, the short half-life of this marker prevents the widespread use of this technology. Meanwhile PET is the technology which can be used to asses tumor tissue hypoxia. The first marker was 2-nitroimidazole and (^18F^)FMISO later it was developed further into ^123^I-tracers. It is an alternative to use reduced chelated metals such as ^60^Cu-compound ATSM although their sensitivity is lower as compared to FMISO. Last but not least, FDG-PET can also be used to assess the glycolysis and increased glucose transport in cancer tissues. Although the specificity of FDG-PET is lower compared to FMISO. The parallel use of the two technologies give the best assessment of tumor tissue hypoxia in clinical situations [56, 57].

## Modulation of Efficacy of Chemo- and Radiotherapy by Tumor Hypoxia

One major modality of cancer treatments is the cytotoxic chemotherapy. However, in a significant proportion of cases, tumors are resistant or acquire resistance during therapy. Chemotherapy resistance depends on genetic and epigenetic factors among which tissue hypoxia is a significant factor. In hypoxia, tumor cells intend to leave cell cycle, and the apoptotic processes are inhibited–these result in decreased sensitivity to cell proliferation blocking cytotoxic agents. Furthermore, in cancer cells, hypoxia induces drug transporter proteins promoting their chemoresistance. At first, in hypoxia HIF1α induces MDR1/ABCB1 efflux transporter resulting in resistance to chemotherapeutics which are its substrates (like doxorubicin)–this mechanism is quite universal among various cancer types [58]. On the other hand, in hypoxia oxygenic stress response is activated by NRF2, which activates HIF1α, but more importantly an array of multidrug resistance genes such as *MDR1/ABCB1, MRP1/ABCC1* and *BCRP/ABCG2* resulting in resistance to a variety of other chemotherapeutics [59, 60].

Furthermore, tumor tissue is characterized by perfusion hypoxia due to the abnormal structure of the intratumoral blood vessels which are also incompetent delivering cytotoxic drugs. Even some chemotherapeutics require O_2_ for optimal effects [1, 61]. It is of note, chemotherapy resistance can be predicted by the expression of HIF1α in some types of squamous cancers [1].

Other major therapeutic modality of cancers is radiotherapy, however, it requires optimal normoxic conditions [62]. Oxygen enhancement ratio refers to the enhancement of the therapeutic effect of irradiation due to the presence of oxygen. Ionizing radiation induces DNA damages by free radicals which are stabilized by ROS. In hypoxia, ROS production is decreased and intracellular SH-containing molecules (glutathione and cysteine) “repair” DNA damages by back-reducing free radicals in DNA. Hypoxia-induced cell cycle arrest and apoptosis resistance (by BCL2 overexpression) decrease the sensitivity of tumor tissue to irradiation. HIF1α overexpression in oral cancer is a negative predictive factor for radiotherapy [63]. For the maximal efficacy of radiotherapy, it is important to induce endothelial cell apoptosis as well. However, the elevated VEGF level promotes endothelial cell survival in hypoxic microenvironment. Accordingly, the alteration of tumoral microvessel density upon irradiation is a sensitive prognostic factor for radiotherapy efficacy [64]. Meanwhile these effects of hypoxia are unique to X-ray irradiation and much less pronounced in other radiotherapy modalities. On the other hand, fractionated irradiation improved the antitumoral effects due to the better timing of irradiation for the reoxygenization period in the tumor tissue.

## Anti-Angiogenic Therapy of Cancer

It has been considered that the inhibition of tumor-induced angiogenesis could have potential antitumoral effects [1]. Considering widespread effects of hypoxia, it would be irrational to deepen hypoxia further in tumor tissues. Overall, anti-angiogenic drugs have been developed and this therapy became the fourth modality following chemo-, radiotherapy and surgery. There are two major groups of anti-angiogenic drugs, the anti-VEGF agents (mostly antibodies) and small molecular inhibitors of VEGFR [65, 66] (Table 3). Although in preclinical models these drugs were able to decrease tumoral microvessel densities – this has never been demonstrated at clinical circumstances. Later on, it was turned out that all these agents are able to normalize the malfunctioning tumoral blood vessels [67] by improving tumor tissue perfusion and decreasing hypoxia.

**TABLE 3:** Clinical use of antiangiogenic drugs [33, 34].

	Drug type	Molecular target	Clinical use
Ligand inhibitors			
Bevacizumab	Monoclonal antibody	VEGF-A	RCC, GBL, OEC, CRC, LUAD, CeC, BC
Ziv-Aflibercept	Recombinant peptide	VEGF-A/B, PIGF, VEGF-C/D	CRC
Receptor inhibitor (ECD)			
Ramucirumab	Monoclonal antibody	VEGFR2	CRC, LUAD, GaC
Kinase inhibitors			
Sunitinib	Small molecular inhibitor	VEGFR1/2/3 PDGFRβ, KIT, RET	RCC
Sorafenib	„	VEGFR1/2/3 PDGFR, KIT, RET, RAF	RCC, HCC
Pazopanib	„	VEGFR1/2, FGFR, KIT	RCC, STS
Axitinib	„	VEGFR1/2/3	RCC
Regorafenib	„	VEGFR, PDGFR, FGFR, TIE2, RAF, KIT	CRC, HCC
Cabozantinib	„	VEGFR, TIE2, MET, RET	RCC, HCC

BC, breast cancer; CeC, cervical cancer; CRC, colorectal cancer; ECD, extracellular domain; GaC, gastric cancer; GBL, glioblastoma; HCC, hepatocellular carcinoma; LUAD, lung adenocarcinoma; OEC, ovarian epithelial cancer; RCC, renal cell cancer; STS, soft tissue sarcoma.

On the other hand, these agents are not effective in monotherapy except for renal cell cancer. This cancer is multidrug resistant, it genetically depends on HIF activation and characterized by extreme VEGF production. This genetic hypoxia dependence is due to the frequent loss of function mutations of VHL [13]. In any other cancer types, anti-angiogenic dugs are effective only in combination with chemo- and/or radiotherapy i.e. increasing the efficacy of cytotoxic therapies by decreasing tissue hypoxia. It is another fact that anti-angiogenic drugs are not effective in combinations with other targeted therapies, with the exception of *EGFR*-mutant lung adenocarcinoma where EGFR inhibitors can be effectively combined with anti-VEGF antibody [68].

It is an equally important question whether anti-angiogenic agents can be effective in cancers where the driver oncogene induces constitutive HIF activation. In case of colorectal cancers, chemotherapy in combination with anti-VEGF antibody is similarly effective in *KRAS*-mutant and wild-type tumors [69]. On the contrary, in case of lung adenocarcinoma, chemotherapy combination with anti-VEGF antibody is effective in *KRAS* wild-type tumors exclusively [70].

It is a further issue if the efficacy of anti-angiogenic agents depends on the type of tumor vascularization or not. Looking into the indications of these anti-angiogenic agents [65, 66] (Table 3), there are tumors where 1) neo-angiogenesis is predominant (renal, breast and colorectal cancers), 2) vessel cooption is characteristic (glioblastoma, lung adenocarcinoma) and 3) unique vascularization form can be observed (liver or esophageal cancers).

However, similar to almost all cancer treatment forms, anti-angiogenic therapy also leads to emergence of resistance. One possible cause of anti-angiogenic therapy resistance is the switch of the angiogenic phenotype: VEGF to PDGF in renal cell cancer, VEGF to FGF in squamous cancers, VEGF to Bv8 peptide in glioblastoma, VEGF to TGFβ in hepatocellular cancer [71], and VEGF to apelin in lung or breast cancers [72]. Similar to other therapeutic modalities, efficacy of anti-angiogenic agents is also dependent on the optimal perfusion of the tumor tissue [73].

## Effect of Hypoxia on the Efficacy of Immunotherapy

If hypoxia affects antitumoral immune responses, it is justified to propose that hypoxia may affect immunotherapy as well [74]. Unfortunately, there are scanty experimental data in this respect, however, clinical developments may help to answer this important question. It can be reasonable to propose that in case of a tumor type where anti-angiogenic therapy is effective, it can be further improved by immunotherapy. It has become evident, that only certain patients and tumors respond well to immunotherapy. This can also suggests to combine immunotherapy with anti-angiogenic agents to decrease hypoxia and VEGF levels in such cases. It is also an important consideration that a fraction of anti-angiogenic agents are “dirty” (not highly specific) VEGFR inhibitors which affect other receptors, crucially important in the normal function of anti-tumoral T-cells. Accordingly, consideration of “pure” (more specific) VEGFR blockers may have a higher chance for clinical efficacy. In case of renal cell cancer, anti-angiogenic monotherapy is the basis of tumor management. The development of combination strategies with immunotherapy was clinically very effective [75] leading to FDA approval of several combinations with multikinase inhibitors (Table 4). Immunotherapy and anti-angiogenic treatment combination also approved recently in lung adenocarcinoma [76] and hepatocellular carcinoma [77] (Table 4). Furthermore, in various cancer types such as colorectal, ovarian or breast cancers, anti-angiogenic treatment (anti-VEGF antibody) and anti-PD1 antibody therapies are approved individually, accordingly it is expected that such combinations will also be a part of clinical management of patients soon.

**TABLE 4:** Approved combinatorial therapies of anti-angiogenic agents and immune checkpoint inhibitors.

Tumor	Anti-PD1 Ab	Anti-PDL1 Ab	Anti-VEGF Ab	Anti-angiogenic TKi	Combination approval
LUAD	—	Atezolizumab	Bevacizumab	—	+
HCC	—	Atezolizumab	Bevacizumab	—	+
RCC	Pembrolizumab	—	—	Axitinib	+
	Nivolumab	—	—	Axitinib	+
	—	Avelumab	—	Cabozantinib	+

Ab, antibody; HCC, hepatocellular carcinoma; LUAD, lung adenocarcinoma; RCC, renal cell carcinoma; TKi, tyrosine kinase inhibitor.

## Therapy of Systemic Hypoxia

The primary cause of systemic hypoxia in cancer patients is anemia (low Hgb levels), accordingly it has to be managed to improve efficacies of other therapeutic modalities. However, targeted therapy has to be applied even in case of systemic hypoxia. There are three therapeutic options for anemia: iron supply, transfusion and erythropoiesis stimulating agents (ESA). Unfortunately, transfusion and ESA administration have a severe adverse effect which is thromboembolism. Iron deficient anemia is a frequent cause for cancer patient’s anemia; therefore, beside determination of the Hgb levels, it is necessary to determine Se-ferritin levels and saturation. In case of absolute iron deficiency, iron administration is necessary, in case of relative iron deficiency, iron supply have to be completed with ESA [78, 79]. It is important to mention that beside severe possible side effects of ESA, this treatment may have other important biological effects: normalization of tumoral blood vessels improving drug perfusion [80] or promoting efficacy of radiotherapy [81], asx observed in preclinical models. However, it is assumed that correction of systemic hypoxia may not be equally effective in tumors where the O_2_-independent HIF activation takes place.

## Molecular Therapy of Hypoxia

It was shown above that hypoxia signaling in cancer is a key regulatory pathway affecting several aspects of cancer biology offering an obvious target for intervention. It would be an indirect approach to disconnect hypoxia signaling since the effector of the O_2_-independent oncogenic driver-driven pathway is mTOR (Table 5; Figure 3). In this respect, it has to be mentioned that the first mTOR inhibitor therapies were introduced into the clinic long time ago in case of *VHL*-mutation dependent renal cell cancer (everolimus and tensirolimus), and more recently in case of breast cancer recently [82].

**TABLE 5:** Targeted therapies of HIF in cancer.

Mechanism	Target	Agent	Preclinical	Clinical	Tumor Type
HIFα RNA expression	HIF1α	Antisense	+	−	Various
	HIF2α	sh-RNA	+	−	Various
	HIF1α	ZnSO_4_	+	−	Melanoma
HIFα protein synthesis	HIF1α	Digoxin	+	−	Various
	HIF2α	2-ME	+	−	Various
		Topotecan	+	−	Various
HIFα stabilization	HIFα	HSP-90 inhibitor	+	+	BRC
	mTOR	Everolimus	+	+	RCC
		Temsirolimus	+	+	BRC
Direct HIFα inhibitors	HIF1α	Acryflavine	+	−	Various
		YC-1	+	−	Various
	HIF2α	PT2385	+	+	Various
	HIF2α	MK6482	+	+	VHL syndrome related tumors
DNA binding	HRE	Echinomycin	+	−	Various

BRC, breast cancer; HIF, hypoxia-inducible factor; HRE, HIF-responsive element; RCC, renal cell cancer; sh-RNA, short hairpin RNA; VHL, von-hippel lindau.

Grey shade: FDA approvals.

In experimental models, it was possible to downregulate HIF1/2α by antisense oligos or sh-RNA [83]. In human melanoma preclinical models, ZnSO_4_ administration was able to downregulate HIF1α expression selectively which had antitumoral and antimetastatic effects [84]. The other approach is to target HIF protein synthesis using digoxin [83], 2-methoxy-estradiol [85] or topotecan [83]. According to preclinical models, these agents could be effective, however, there are no available clinical data. Stabilization of HIFα can be suspended by HSP-90 inhibitor *in vivo* [86].

Of note, the HIF complex-induced binding to HRE regions in promoters of various genes can be achieved by echinomycin [87]. However, the ultimate goal have to be the development of small molecule HIF inhibitors. *In vitro*, acriflavin [83] or YC-1 [88] can directly bind to HIF1α; although they have shown some preclinical activity, the clinical development was discontinued. However, small molecule HIF2α inhibitors, PT2977/MK6482 and PT2385 have been developed recently and tested in clinical trials [89]. MK6482 was used clinically in VHL syndrome related renal cell cancer with promising activity [90]. Based on these results, MK6482 became the first FDA-approved HIF2α inhibitor.

Anti-hypoxia therapies have already been introduced in case of radiotherapy using hyperbaric O_2_ (HBO), which could have some activity in squamous cancers of the head and neck [91]. It is another clinical approach to combine accelerated radiotherapy with nicotinamide and carbogen (ARCON) of which research reached phase-III [92] but did not resulted in clinical acceptance. Another clinically active therapeutic option is “chemical anti-hypoxia” by the use of doranidazole or nimorazol in lung cancer radiotherapy [93] which are used more widely nowadays due to the success of the DAHANCA 5–85 trial. Generation of intratumoral O_2_ is also feasible to improve the efficacy of radiotherapy by using bioactive albumin-MnO_2_ nanoparticles [94]. The imminent question concerning all these approaches is whether any of these could be exploited in case of other anticancer therapeutic modalities such as chemotherapy, targeted therapy, anti-angiogenic therapy or immunotherapy.

## Metabolic Therapy

**FIGURE 6:**
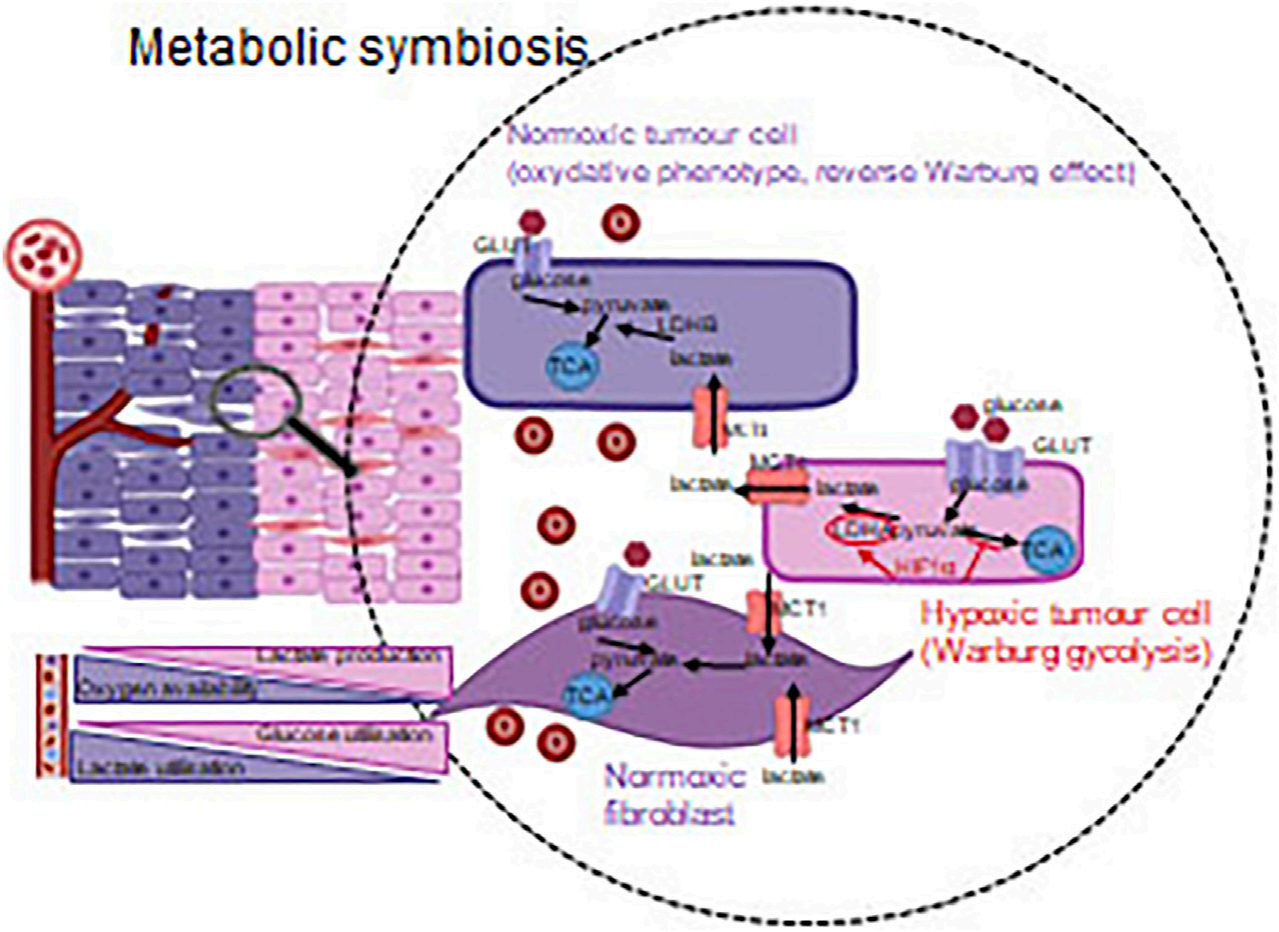
Metabolic symbiosis–optimizing the available energy sources. Tumorous and other non-tumorous cells derived from microenvironment utilize the nutrients in harmony with the oxygen concentration (via the regulating role of HIF1α). Accordingly, not only the glycolysis, but also the reverse Warburg effect–in a well-oxygenated environment–provide adaptation capacity/opportunity for cancerous cells.

During tumor progression, angiogenic and metabolic effects of HIF activation are in a complex relation, and crosstalk among the related signaling machineries can be important. Metabolic adaptation can be observed in case tumor cells survive the consequences of anti-angiogenetic or anti-HIF1α treatments. This phenomenon can be illustrated with the example of metabolic symbiosis and heterogeneity [95]. Tumor cells in the tissue context, optimize and balance the utilization of the available energy resources to maintain their continuous proliferation even in stress conditions. In oxygenated environment, tumorous or even normal cells can consume the excreted metabolites of other cells located in the stroma. As an example, lactate produced during “lactic glycolysis” of hypoxic or pseudo-hypoxic cells can be utilized in this manner [37] (Figure 6). Metabolic plasticity and tissue co-operation are important factors of the tumor resistance to various therapies. Metabolic adaptation also has a remarkable role in maintaining/supporting the survival of the so-called cancer stem- or dormant cells [96, 97].

All of these provide an opportunity for therapeutic exploitation of targeting metabolic symbiosis, which can also lead to the introduction of novel therapeutic options in anti-angiogenic combination treatments. It was observed that the activity pattern of mTOR shows intratumoral heterogeneity as a consequence of anti-angiogenic therapies. This finding calls the attention to the master regulatory role of mTOR kinase in developing therapy resistance [98]. Several clinical trials of renal carcinoma, glioma, neuroendocrine and gastrointestinal cancers are ongoing, involving mTOR inhibitors in combination with anti-angiogenic treatments, however, the results may vary between tumor types [99–102]. Beside mTOR inhibitors, additional opportunities can be found for the inhibition of metabolic adaptation and symbiosis. The uptake and the release of extracellular metabolites and their transporters can also be inhibited in these metabolic alterations. Moreover, various metabolite transporter proteins–involving monocarboxylate transporters (MCTs), which contribute to lactate transport–can also be tested as a part of anti-angiogenic treatment combination [38, 39, 103, 104].

Autophagy can be induced by certain treatments or microevironmental effects in relation to metabolic adaptation and resistance [45]. It can either result in apoptosis of cancer cells or provide appropriate bioenergetic background for cellular survival. Autophagy activation can be observed in case of anti-angiogenic therapies. It is not surprising that autophagy inhibiting/inducing factors could be associated with anti-tumor effects in certain combinational treatments. Autophagy-targeted therapies and the combinatorial effect of anti-angiogenic treatments were confirmed among experimental conditions e.g. in lung carcinomas [105].

As a further metabolic mechanism, mitochondrial oxidation cannot be neglected as a potential therapeutic target (i.e. metabolic phenotype of cancer stem cells or tumor cells in an oxygenized environment). The anti-diabetic drug, complex I inhibitor (OXPHOS) metformin and phenformin (AMPK activator) can inhibit mitochondrial electron transport chain. Based on certain studies, their combination with chemotherapy could be effective, but it could significantly enhance the effect of anti-angiogenic therapy (bevacizumab) without increasing the severity of side effects (e.g. in metastatic NSCLC) [105–107]. Other studies also call the attention to metabolism-targeting agents in combinations, which highlights that these could potentially enhance the impact of sensitizing strategies and accordingly, mTOR inhibitors could inhibit tumor progression as having a complex modifying role on metabolism [108–113].

Treatments (including anti-angiogenic ones) targeting metabolic adaptation mechanisms and influencing metabolic symbiosis administered in combination could cause metabolic catastrophe in cancer tissue adaptation machinery. These could also help the development of traditional targeted or anti-angiogenic therapy combinations. However, tumor heterogeneity, similarly to immunoediting mechanisms, always has to be considered in experimental model systems [114, 115]. Significant differences could be obtained by studying *in vitro* and *in vivo* models or clinical tumor specimens. Therefore, the administration of metabolic inhibitors has to be verified a priori of clinical trials, and appropriate biomarkers have to be discovered to use them in a “targeted” or precision manner.

## Conclusion

Our knowledge in the past decades enormously increased abut the mechanism and detriemental consequences of tumor hypoxia. Fortunately, these information led to development several new therapeutic modalities and started to transform the use of existing therapies. However, the tumor hypoxia issue must be developed into a core aspect of cancer management from diagnosis through treatment to effective cure.

## Abbreviations

EPO, erythropoietin; ESA, erythropoiesis stimulating agent; FDG-PET, fluoro-deoxy-glucose positron emission tomography; FMISO, fluoromisonidasole; HBO, hyperbaric O2; Hgb, hemoglobin; HIF, hypoxia inducible factor; HRE, HIF-responsive element; OXPHOS, oxidative phosphorylation, ROS, reactive oxygen species; TCA, tricarboxylic acid cycle; VEGF, vascular endothelial growth factor; VHL, von Hippel Lindau.
